# Research into the Strength of an Open Wagon with Double Sidewalls Filled with Aluminium Foam

**DOI:** 10.3390/ma14123420

**Published:** 2021-06-20

**Authors:** Oleksij Fomin, Mykola Gorbunov, Juraj Gerlici, Glib Vatulia, Alyona Lovska, Kateryna Kravchenko

**Affiliations:** 1 Department of Cars and Carriage Facilities, State University of Infrastructure and Technologies, Kyrylivska Str., 9, 04071 Kyiv, Ukraine; fomin1985@ukr.net; 2 Department of Railway, Automobile Transport and Handling Machines, Volodymyr Dahl East Ukrainian National University, Central Avenue 59a, 93400 Sewerodonetsk, Ukraine; gorbunov0255@gmail.com; 3 Department of Transport and Handling Machines, University of Zilina, Univerzitna 1, 010 26 Zilina, Slovakia; juraj.gerlici@fstroj.uniza.sk; 4 Department of Structural Mechanics and Hydraulics, Ukrainian State University of Railway Transport, Feuerbach Square 7, 61050 Kharkiv, Ukraine; vatulya@kart.edu.ua; 5 Department of Wagon Engineering and Product Quality, Ukrainian State University of Railway Transport, Feuerbach Square 7, 61050 Kharkiv, Ukraine; alyonalovskaya@kart.edu.ua

**Keywords:** open wagon with double sidewalls, bearing structure, dynamic stresses, strength, transport mechanics

## Abstract

The research is concerned with the use of double walls filled with aluminium foam for an open wagon in order to decrease the dynamic stresses during the operational modes. The research presents the strength calculation for the bearing structure of an open wagon with consideration of the engineering solutions proposed. It was found that the maximum equivalent stresses appeared in the bottom section of the centre sill behind the back support; they amounted to about 315 MPa and did not exceed the allowable values. The maximum displacements were detected in the middle section of the centre sill and amounted to 9.6 mm. The maximum deformations were 1.17 × 10^−2^. The research also presents the strength calculation for a weld joint in the maximum loaded zones of the bearing structure of an open wagon and gives the results of a modal analysis of the bearing structure of the improved open wagon. It was found that the critical oscillation frequencies did not exceed the allowable values. The results of the research may be useful for those who are concerned about designing innovative rolling stock units and improving the operational efficiency of railway transport.

## 1. Introduction

Higher operational efficiency of transportation can be ensured by introducing innovative design solutions for modern transportation facilities [1]. One of the most promising industries is railway transport which requires introduction of new engineering solutions for the bearing structure of a rail car in order to maintain a leading place in the transportation market. These solutions will reduce the material capacity of freight wagons, increase the loading capacity and the average speeds of both loaded and empty freight wagons, improve anticorrosion and antifriction properties of the construction materials, prolong the service life, and decrease the total production costs and operational expenditures. All of these may increase the operational efficiency of railway transport and improve its competitiveness both nationally and internationally.

## 2. Analysis of Recent Research and Publications

The research into the fatigue strength of an open wagon body during operational loading modes is presented in [2,3]. The research was made for an open wagon C80B. The authors found that it is possible to predict the strength capacity of a body with the hybrid dynamic modelling and analysis by means of the finite element method.

The theoretical and experimental research into the strength capacity of a freight wagon is given in [4]. The study included the normative operational loads on the wagon relative to the rail tracks. However, the authors did not study the issue of how to decrease the material capacity and dynamic loads on the bearing structure of an open wagon in operation. At the same time, tests were carried out for vertical loading and horizontal compression. The test results made it possible to determine the localisation of stress distribution zones in the load-bearing structure of the wagon. However, the research does not consider the prospects for improving the load-bearing structure of the wagon to increase the efficiency of its operation.

However, the authors did not study the issue of how to decrease the material capacity and dynamic loads on the bearing structure of an open wagon in operation.

The peculiarities of the structural and parametrical optimization of a wagon are studied in [5]. The evolutionary optimisation diagram of stem, plate, and plate–stem elements was conducted by the algorithm which selected variants of the bearing system by means of the target function criteria. Additionally, the authors considered random changes in the parameters and an exchange of parameters in the variant pairs for the bearing structure.

Along with this, the authors do not conduct a study of the dynamic stresses of the proposed car design, and when compiling the design schemes, the standard values of the loads are taken into account. In addition, the paper does not consider the possibility of using materials with damping properties to reduce the dynamic stresses of the wagon in operation.

The optimization of open-type freight wagon bodies with the bearing floor is given in [6]. The authors developed a combined algorithm of structural and parametrical optimisation for the side wall and the frame of an open wagon with the bearing floor intended for an axial load on 25 t/axle. However, the main design loads were taken as a quasi-static load in accordance with the normative documentations. Therefore, the authors did not determine the actual dynamic loads on the optimised wagon structure. At the same time, an increase in the axle load entails an increase in the dynamic stresses of the bearing structure, which necessitates the use of draft gear with increased energy consumption or the introduction of damping materials into the bearing structure. However, such studies are absent in this work.

Computational modelling and simulations are nowadays widely used for design of railway vehicles, analysis and assessment of their dynamic properties, and also for detection and prediction of the mechanism of deterioration and sources of damages. In [7] (p. 1), the process of computational modelling of a freight wagon multibody system is outlined. The results of the dynamic stresses modelling of a wagon during movement are presented. The actual values of the dynamic loads acting on the bearing structure of the wagon were determined. The results obtained made it possible to formulate the requirements for the design of the modern rolling stock. However, the authors of the article did not carry out studies aimed at reducing the dynamic stresses of the bearing structure under operating conditions.

The concept of designing a car body of aluminium panels is considered in [8]. A special feature of such panels is their sandwich-type structure. The characteristic search function for the optimal combination is based on the maximum stresses and displacements. However, the authors did not investigate the possibility of using damping material for the frame and beam elements of a wagon, as the most loaded elements of the carriage supporting structure.

The special aspects of modernization for freight wagon bodies are considered in [9]. The authors proposed some measures for prolonging the service life of a wagon and improving the diagnostic systems for the technical state of the modernized wagon bodies. To increase the time between repairs of the wagon, the authors proposed strengthening the structure in the interaction zones of the constituent elements, as well as the use of anti-corrosion coatings. However, this modernization is aimed at strengthening the load-bearing structure of the wagons. At the same time, the possibility of using materials with damping properties to reduce the dynamic stresses of the wagon in operation is not considered.

Study [10] deals with the topological optimization of a wagon body on the basis of the finite element method. The results of the research confirmed the efficiency of the technique proposed for a wagon body. It was found that the considered technique turned out to be an effective tool for obtaining an optimal thin-walled structure. However, the authors did not study the dynamic stresses of the bearing structure. Due to the fact that the optimized design has a lower mass, then in operation, it will experience a greater dynamic load. This can influence its strength. In this connection, it is advisable to consider issues aimed at reducing the dynamic stresses of the bearing structure in operation. However, the authors of the article did not pay attention to this.

## 3. Purpose and Tasks of the Research

The purpose of the research was to demonstrate special features of the strength calculation for the bearing structure of an open wagon with double sidewalls with filler during operational loading modes. To achieve the objective the following tasks were set:

− To design the bearing structure of an open wagon with double sidewalls with filler (e.g., aluminium foam) in the program software;
− To calculate the dynamic stresses of the open wagon bearing structure with double sidewalls filled with aluminium foam;
− To calculate the strength of the bearing structure of an open wagon with double sidewalls with filler;
− To calculate the strength of a weld joint in the contact area between the intermediate post and the cross bearer of an open wagon; and
− To conduct a modal analysis of the bearing structure of an open wagon with double sidewalls with filler.

## 4. Presentation of the Main Content of the Article

**Figure 1:**
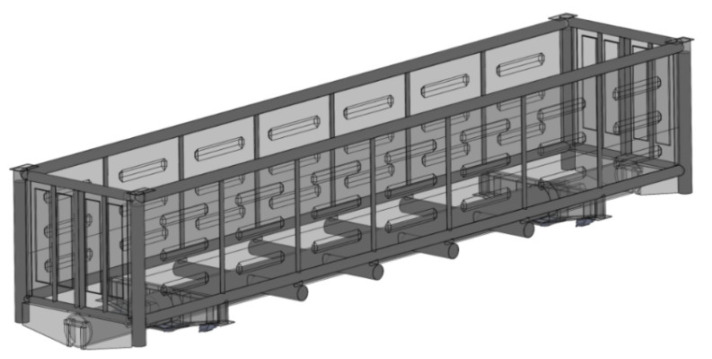
Bearing structure of an open wagon of circular pipes filled with aluminium foam.

**Figure 2:**
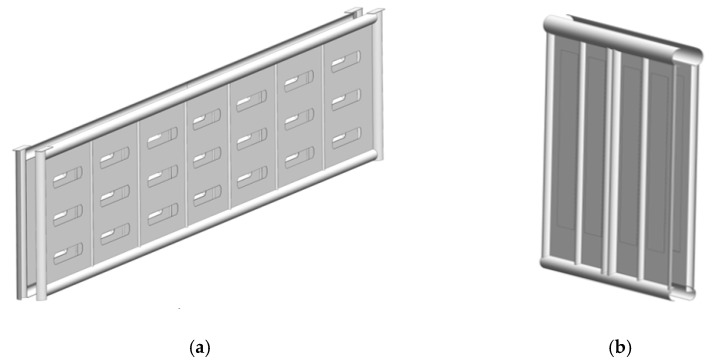
Components of the bearing structure of an open wagon of double walls (**a**) side wall; (**b**) end wall.

The authors proposed the use of circular pipes for the bearing structure in order to decrease the material capacity of an open wagon. A 12-757 open wagon manufactured by Kryukovsky Railway Car Building Works (Ukraine) was taken as the prototype. The authors explored the possibility to achieve a higher rigidity of the bearing structure of an open wagon of circular pipes filled with aluminium foam (Figure 1). The sidewalls and the end doors were doubled (Figure 2). This solution is proposed at the concept level, i.e., scientific idea in order to consider the feasibility of using aluminium foam in wagon structures. Therefore, the issues of determining the optimal pore size and the technology of filling pipes with aluminium foam are not considered in the work.

To substantiate the proposed solution, the dynamic stresses of the open wagon with a filler was determined. At the same time, a mathematical model was formed (1)–(3).

(1) $$\left(M_{W}+2\cdot m_{b}+\frac{n\cdot I_{WS}}{r^{2}}\right)\cdot {\ddot{x}}_{W}+M_{W}\cdot h\cdot {\ddot{\varphi }}_{W}=P-c\cdot {\dot{x}}_{W},$$

(2) $$I_{W}\cdot {\ddot{\varphi }}_{W}+M^{\prime }\cdot {\ddot{x}}_{W}-g\cdot \varphi_{W}\cdot M^{\prime }=l\cdot F_{FR}\left(sign{\dot{\Delta }}_{1}-sign{\dot{\Delta }}_{2}\right)+l\left(k_{1}\cdot \Delta_{1}-k_{2}\cdot \Delta_{2}\right);$$

(3) $$M_{W}\cdot {\ddot{z}}_{W}=k_{1}\cdot \Delta_{1}+k_{2}\cdot \Delta_{2}-F_{FR}\left(sign{\dot{\Delta }}_{1}-sign{\dot{\Delta }}_{2}\right);$$

$$\Delta_{1}=z_{W}-l\cdot \varphi_{W};\;\;\;\;\Delta_{2}=z_{W}+l\cdot \varphi_{W};$$

$M_{W}$—mass of carrying structure of the wagon; $I_{W}$—inertia moment of wagon relative to the longitudinal axle; *p*—value of longitudinal impact force to automatic coupler; c—stiffness of the aluminium foam, $m_{b}$—bogie mass; $I_{WS}$—inertia moment of a wheelset; r—radius of the mean worn-out wheel; n—number of bogie’s axles; l—half-base of wagon; $F_{FR}$—absolute value of dry friction force in spring group; $k_{1}$, $k_{2}$—rigidities of springs in bogie’s suspension; $x_{W}$, $\varphi_{W}$, $z_{W}$—coordinates corresponding to longitudinal, angular around longitudinal, and vertical displacements of the wagon, respectively.

The studies were carried out in a flat coordinate system. The case of a shunting collision was taken into account. At the same time, it was taken into account that a force of 3.5 MN acts on the end stop of the automatic coupler. When solving Equations (1)–(3), it was assumed that the wagon was based on a bogie type 18–100. The starting conditions were taken to be zero. Differential equations were solved in the MathCad software package, which implements the Runge–Kutta method.

It was found that the maximum acceleration of the open wagon bearing structure at the centre of mass was 34.6 m/s^2^, which was 3% lower than the accelerations obtained for the structure of round tubes without filler and 6% lower than the accelerations in the standard wagon design.

The strength of the bearing structure of an open wagon with consideration of the proposed engineering solutions was determined in the CosmosWorks software [11,12,13,14,15] by means of the finite element method. The optimal number of elements in a mesh was determined with the graphic analytical method [16,17,18,19,20,21]. Spatial isoparametric tetrahedrons were used as the finite elements; the optimal number of the elements was defined with the graphic analytical method. The number of units in the mesh was 266,693, and the number of elements was 1,173,216. The maximum element size equalled 70 mm; the minimal element size was 14 mm. The minimum number of elements in a circle was 13; the element size gain ratio in the mesh was 1.8. The maximum side ratio was 596.61; the percentage of elements with a side ratio of less than three was 61.4 and more than ten was 8.13.

**Figure 3:**
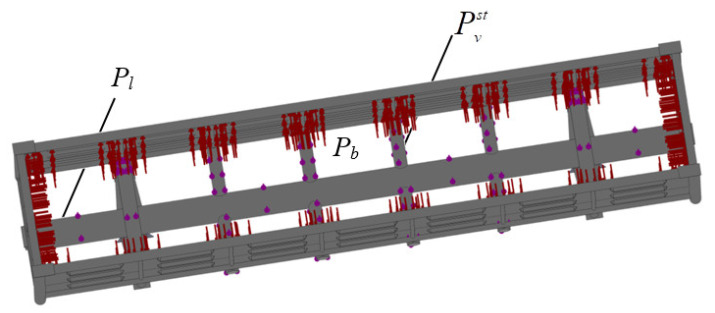
Design diagram of an open wagon: $P_{l}$—the longitudinal force, $P_{v}^{st}$—the vertical static load, $P_{b}$—the bulk freight.

The design diagram included the vertical static load $P_{v}^{st}$ on the bearing structure of an open wagon, the longitudinal force $P_{l}$ on the back support of an automatic coupler, and the pressure from the bulk freight $P_{b}$ on the side and end walls (Figure 3). The lateral pressure from the bulk freight was calculated by the technique given in [22]. Mineral carbon was taken as the bulk freight.

Steel 09G2S was taken as the material for the bearing structure of a wagon. The model was secured in the areas of support on the gear parts. 

The maximum equivalent stresses appeared in the bottom section of the centre sill behind the back support; they amounted to about 315 MPa and did not exceed the allowable values [23,24,25]. The maximum displacements were detected in the middle part of the centre sill; they amounted to 9.6 mm. The maximum deformations were 1.17 × 10^−2^.

The calculation demonstrated that when aluminium foam was used as filler for the centre sill, the maximum equivalent stresses in the bearing structure of an open wagon decreased by about 8%, and displacements decreased by 9% in comparison with the similar values for the circular-piped bearing structure of an open wagon without filler. The mass of the bearing structure of an open wagon increased by 24% in comparison with that of the filler-free structure (at an aluminium foam density of 300 kg/m^3^).

In order to reduce the sprung mass of the wagons, it is advisable to use aluminium foam in the most loaded areas of the supporting structure—namely, the centre beam, end beam, and pinching nodes of vertical struts with cross beams of the frame. At the same time, the bearing structure mass of the wagon will increase by 3.4% in comparison with the structure of pipes without filler. It is important to say that the introduction of round pipes as load-bearing elements of the open wagon provides a 6% reduction in material consumption compared with a prototype wagon. Consequently, taking into account the use of aluminium foam, the mass of the wagon’s load-bearing structure is 2.6% lower than the standard one. In addition, the advisability of using aluminium foam is to reduce the dynamic stresses of the bearing structure due to the damping properties of the filler.

**Figure 4:**
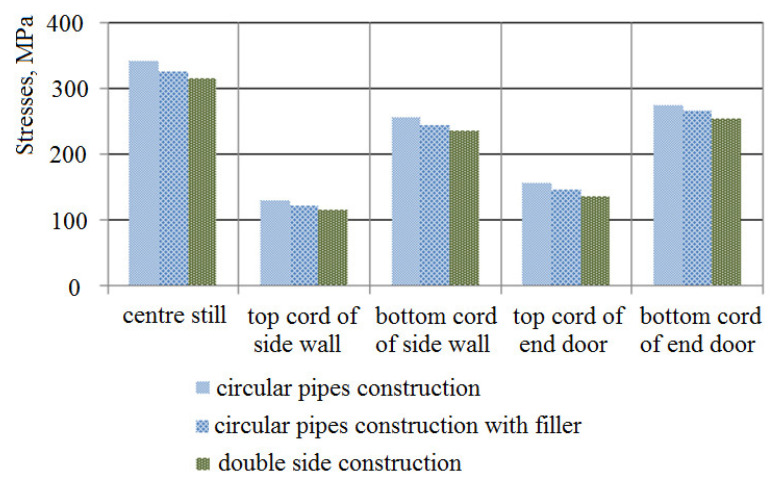
Stresses on components of the bearing structure of a circular-piped wagon.

Figure 4 demonstrates the comparative analysis of the stresses in the bearing structure of an open wagon of circular pipes (I), of circular pipes with filler (II), and of circular pipes and the double side and end walls with filler (III).

The data presented in Figure 4 demonstrate that the minimum stresses on the bearing structure of an open car were detected for variant III. The research shows that the application of filler for the circular-piped bearing structure decreases the operational loading.

**Figure 5:**
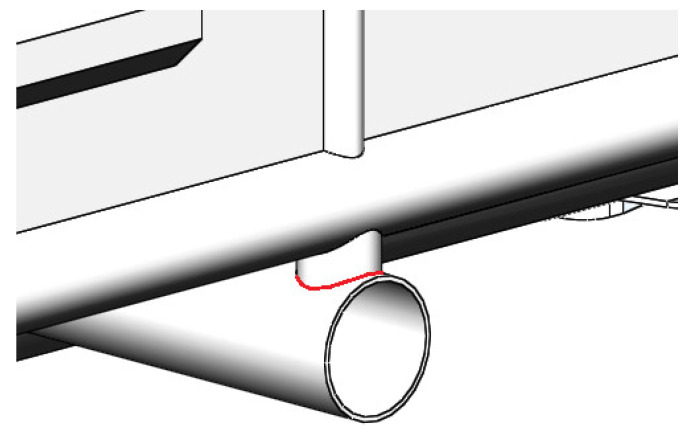
Contact assembly between the intermediate vertical post and the cross bearer of an open wagon frame.

The strength of the improved bearing structure of an open wagon body was studied through determination of the strength of a weld joint in the contact areas of the most loaded elements. The stress strain state calculation for the bearing structure of a wagon body demonstrated that one of the most loaded areas is the contact assembly between the intermediate vertical brace and the cross bearer welded to each other (Figure 5).

Since the proposed bearing structure of the open wagon consists of circular cross-section pipes, then when calculating the welding seams in the most loaded zones, the technique given in [26,27] was used. In addition, this method was chosen due to the fact that standard regulatory documents for the design and calculation of wagons do not regulate such loading modes, since specialized profiles are used as load-bearing elements of the bodies.

The strength of the bearing structure of an open wagon body was calculated through the strength of a weld joint in the contact area between the intermediate vertical post and the cross bearer of an open wagon.

In operation, this assembly suffers the following deformations: tension/compression, bending, and shearing.

According to [26,27], the butt joints of piped elements for the central tension/compression were calculated by the formula:

(4) $$\frac{N}{\pi \cdot D_{m}\cdot t}\leq R_{wy}\cdot \gamma_{wc},$$

where $D_{m}$—average diameter of a pipe with a less wall thickness, mm; t—least wall thickness of pipes connected, mm; $R_{wy}$—design resistance of a weld joint, kPa; $\gamma_{wc}$—coefficient of the condition load effect for a weld joint.

The calculation included the following parameters: $D_{m}=73.25\;\mathrm{mm}$, t=5.5 mm, $R_{wy}$ was calculated according to [27] and amounted to 328.57 × 10^−3^ MPa, $\gamma_{wc}=0.75$. The longitudinal force value was determined with the assumption that the pressure force to the cross bearer of an open wagon body created a static impact and acted as the evenly distributed loading. The value of this loading equalled the maximum pressure loading to the post (the triangle foundation). By taking into account the surface of the post located inside the body, the authors determined the longitudinal force to the weld joint in the contact area between the intermediate post and the cross bearer. The calculation demonstrated that condition (4) was fulfilled, and the strength of a weld joint was provided.

Additionally, the authors checked the strength of the weld joint when a circular pipe was attached to another detail (a circular pipe) under the action of the longitudinal force:

(5) $$N\leq 0.85\cdot \left(S_{wh}+S_{wt}\right),$$

(6) $$N\leq 2\cdot S_{wh},$$

(7) $$N\leq 2\cdot S_{wt},$$

where $S_{wh},\;S_{wt}$—bearing capacity of the root of weld and the face of weld defined by the formula:

(8) $$S_{wh}=\left(t_{d}\cdot l_{wah}\cdot R_{wc}\cdot \gamma_{wc}+k_{f}\cdot l_{wfh}\cdot R_{wd}\right)\cdot \gamma_{wc},$$

where $R_{wd}$—corresponds to the numerical value $R_{wf}$ or $R_{wz}$, a lower value is taken for calculation: $0.7\cdot R_{wf}$ or $R_{wz}$, kPa; $R_{wf}$ and $k_{wz}$—design resistance of an angular joint (conditional), respectively, by the weld metal and the weld fusion boundary, kPa; $t_{d}$—thickness of a pipe wall attached, mm; $k_{f}$—height of an angular weld joint, mm; $l_{wah}$ and $l_{wat}$—total length of the joint sections taken as butt joints, in the root of weld and the face of weld, respectively, mm; $l_{wfh}$ and $l_{wft}$—total length of the joint sections taken as angular joints in the root of weld and the face of weld, respectively, mm.

On the basis of the calculation, it was found that the weld joint provided the needed strength. The following data were included in the calculation: *t_d_* = 5.5 mm, *l_wah_* = 1.06 mm, *l_wat_* = 1.12 mm, *k_f_* = 4.5 mm (with consideration of the thickness of the pipe welded and the welding conditions), *R_wd_* = 150.92 × 10^−3^ kPa (at *R_wf_* = 215.6 × 10^−3^ kPa and *R_wd_* = 196 × 10^−3^ kPa).

**Figure 6:**
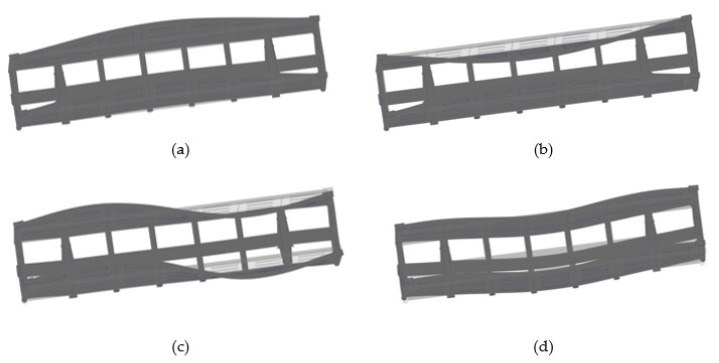
Forms of the oscillations of the bearing structure of an open wagon (scale 20:1) (**a**) 1st natural frequency; (**b**) 2nd natural frequency; (**c**) 3rd natural frequency; (**d**) 4th natural frequency.

The research included a modal analysis of the bearing structure of an open wagon with double walls filled with aluminium foam by means of the design diagram (Figure 3) and conducted in the CosmosWorks software. Some forms of the natural oscillations are given in Figure 6.

The natural oscillations obtained are given in Table 1.

The data presented in Table 1 demonstrate that the natural oscillation frequencies are within the allowable values [23,24]. At the same time, taking into account the use of aluminium foam, the natural vibration frequencies of the structure change by 5% compared with the vibration frequencies of the structure without a filler.

## 5. Conclusions

1. The research shows the development of the bearing structure of an open wagon with double walls with filler (e.g., aluminium foam) in the program software. This solution will increase the rigidity of the bearing structure of an open wagon in operation and decrease the dynamic stresses and vulnerability under the most unfavourable operational modes.
2. The calculation of the dynamic stresses of the bearing structure of the open wagon with double sidewalls filled with filler was carried out. It was found that the maximum acceleration of the open wagon bearing structure at the centre of mass is 34.6 m/s^2^, which is 3% lower than the accelerations obtained for the structure of round tubes without filler and 6% lower than the accelerations in the standard wagon design.
3. By applying aluminium foam as filler for the centre sill, the maximum equivalent stresses in the bearing structure of an open wagon decreased by about 8%, and displacements decreased by 9% in comparison with the similar values for the bearing structure of circular pipes without filler. The mass of the bearing structure of an open wagon increased by 24% in comparison with that of the filler-free structure (at an aluminium foam density of 300 kg/m^3^).
  In order to reduce the sprung mass of the wagons, it is advisable to use aluminium foam in the most loaded areas of the supporting structure—namely, the centre beam, end beam, and pinching nodes of vertical struts with cross beams of the frame. At the same time, the bearing structure mass of the wagon will increase by 3.4% in comparison with the structure of pipes without filler. It is important to say that the introduction of round pipes as load-bearing elements of the open wagon provides a 6% reduction in material consumption compared with a prototype wagon. Consequently, taking into account the use of aluminium foam, the mass of the wagon’s load-bearing structure is 2.6% lower than the standard one.
4. The research presents the strength calculation of a weld joint in the contact area between the intermediate post and the cross bearer of an open wagon frame. The following deformations in such an assembly were taken into account: tension/compression, bending, and shearing. With *R_wd_* = 150.92 × 10^−3^ kPa (at *R_wf_* = 215.6 × 10^−3^ kPa and *R_wf_* = 196 × 10^−3^ kPa), the strength of the weld joint was provided at these deformations.
5. The authors conducted a modal analysis of the bearing structure of an open wagon with double walls with filler and obtained the main forms and the numerical values of the natural oscillation frequencies of the bearing structure of an open wagon. It was found that the natural oscillation frequencies did not exceed the allowable values.
  The research will be useful for those who are concerned about designing innovative rolling stock units and improving the operational efficiency of railway transport.

## Figures and Tables

**Table 1:** Natural oscillation frequencies in the bearing structure of an open wagon.

Form of Oscillations	Frequency, Hz (Filler-Free Construction)	Frequency, Hz (Filled Construction)
1	17.012	16.047
2	17.543	16.661
3	28.567	27.645
4	28.678	28.958
5	30.521	29.738
6	38.654	38.146
7	40.121	39.094
8	42.015	41.921
9	47.656	46.46
10	50.651	49.573

## Data Availability

Not applicable.
